# Long-read low-pass sequencing enhances variant detection in a peanut MAGIC population

**DOI:** 10.1093/g3journal/jkag196

**Published:** 2026-08-05

**Authors:** Kendall Lee, Walid Korani, Sameer Pokhrel, Hallie Wright, Philip C Bentz, Peggy Ozias-Akins, Ye Chu, Alex Harkess, Justin Vaughn, Josh Clevenger

**Affiliations:** HudsonAlpha Institute for Biotechnology, Huntsville, AL 35806, United States; HudsonAlpha Institute for Biotechnology, Huntsville, AL 35806, United States; Institute of Plant Breeding, Genetics and Genomics, Center for Applied Genetic Technologies, University of Georgia, Athens, GA 30602, United States; HudsonAlpha Institute for Biotechnology, Huntsville, AL 35806, United States; HudsonAlpha Institute for Biotechnology, Huntsville, AL 35806, United States; Department of Horticulture, University of Georgia, Tifton, GA 31793, United States; Department of Horticulture, University of Georgia, Tifton, GA 31793, United States; HudsonAlpha Institute for Biotechnology, Huntsville, AL 35806, United States; HudsonAlpha Institute for Biotechnology, Huntsville, AL 35806, United States; HudsonAlpha Institute for Biotechnology, Huntsville, AL 35806, United States; Institute of Plant Breeding, Genetics and Genomics, Center for Applied Genetic Technologies, University of Georgia, Athens, GA 30602, United States

**Keywords:** Long-read sequencing, Low-pass sequencing, Pangenome, Structural variation, Multi-parent population, MAGIC population, Peanut, *Arachis hypogaea*, Variant calling, Trait mapping

## Abstract

Accurate genotyping accelerates crop improvement, yet long-read sequencing remains underused in breeding due to cost. We present a scalable long-read low-pass (LRLP) sequencing framework for high-throughput variant discovery and trait mapping. Using PacBio HiFi reads in an allotetraploid peanut (*Arachis hypogaea*; AABB, 2n = 4x = 40) MAGIC population, we generated both LRLP and short-read low-pass (SRLP) data. At comparable depths, LRLP achieved substantially greater whole-genome and gene–space coverage than SRLP. Data were analyzed using both a single-reference genome and an 18-parent pangenome graph constructed with KhufuPan, a new tool for graph-based genotyping. Across analytical approaches, LRLP consistently identified more SNPs, indels (2–1,000 bp), and structural variants (>1 kb) than SRLP, improving genotype resolution and selection accuracy, particularly for large structural variants. By reducing cost barriers and increasing variant discovery in complex genomes, LRLP provides a practical path for deploying advanced genomics in under-resourced and orphan crops critical to global food security.

## Introduction

The global population is projected to approach 10 billion by 2050 (UN Population Division, 2024). Meeting the caloric needs of this growing population will require an estimated 60% increase in agricultural crop production (Bhatta et al. 2021). Compounding this challenge, global warming has already reduced global crop yields by 21% since the 1960s, presenting additional hurdles for farmers and plant breeders (Ortiz-Bobea et al. 2021). The predicted yield increases for major crops like maize, rice, wheat, and soybean in the coming decades will be insufficient for meeting global food demands (Ray et al. 2013). Addressing these challenges requires rapid crop improvement to enhance yield and productivity while mitigating climate-induced stresses, such as drought and disease.

One significant obstacle in plant breeding is the time required to evaluate new cultivars for desirable traits. However, advances in DNA sequencing technologies over the past decade have paved the way for molecular-based plant breeding. Marker–trait association techniques, such as genome-wide association studies (GWAS) and quantitative trait locus sequencing (QTL-seq), have accelerated the development of new cultivars by enabling marker–trait-based selection (Wang et al. 2017; Ferrão et al. 2018; de Bem Oliveira et al. 2019; Cui et al. 2020; Montanari et al. 2022; Guo et al. 2024; Pandey et al. 2024). These approaches primarily rely on short-read sequencing for trait mapping, which involves statistical analyses to correlate genetic variants with phenotypic traits (Wang et al. 2017; Zhao et al. 2021a; Ayalew et al. 2022). Short-read sequencing remains popular due to its affordability, with low-pass sequencing (∼1x) further driving down costs (Li et al. 2021). Low-pass whole-genome sequencing approaches have demonstrated high SNP (single nucleotide polymorphism) calling accuracy at depths as low as 0.5x in plants (Korani et al. 2021). Khufu, a proprietary single-reference genotyping analysis method (https://www.hudsonalpha.org/khufudata/), applies these principles with additional quality filtering for breeding applications (Bian et al. 2021; Korani et al. 2021; Liu et al. 2024; Li et al. 2025). However, short reads have significant limitations (Schiessl et al. 2019; Amarasinghe et al. 2020). Typically generating read lengths of around 150 base pairs (bp), short reads often align ambiguously to multiple regions of a reference genome, reducing confidence in genotype calls and filtering out potentially informative data. This is especially problematic in complex plant species, such as polyploids. Moreover, short-read sequencing fails to capture large structural variations (SVs) such as insertions, deletions, inversions, translocations, and duplications, which are increasingly recognized as key contributors to agronomic traits (Vaughn et al. 2021; Zhao et al. 2021b; Pei et al. 2024). For instance, SVs have been implicated in important agronomic traits as well as improvements during long-term breeding programs (Stein et al. 2017; Hurgobin et al. 2018; Jung et al. 2019; Guo et al. 2020; Vaughn et al. 2022; Lyu et al. 2023; Zhang et al. 2023).

Long-read sequencing has emerged as a powerful tool for reference genome assembly. It can generate sequences tens of thousands of base pairs in length (Chawla et al. 2021). These longer reads simplify the alignment to reference genomes. They also enable the detection of SVs (Kang et al. 2023). Despite these advantages, long-read sequencing has historically been limited to high-coverage sequencing of a few samples. The main challenges are the high cost and low throughput of wet-lab protocols for extracting high-molecular-weight (HMW) DNA and preparing sequencing libraries (Ebler et al. 2022). Our optimization of LRLP sequencing for plants offers a solution. Pooling multiple samples for low-coverage sequencing with LRLP significantly reduces the cost per sample while preserving the benefits of long reads for genotype calling (Solares et al. 2018). This makes long-read sequencing feasible for routine use in breeding programs.

Another recent advancement in genomics is the pangenome graph. This data structure integrates multiple reference genomes into a unified framework. Traditional single-reference genomes often fail to capture SVs and gene presence/absence variation, which are common in plant species. Pangenome graphs provide a more representative model of species-wide diversity (Ebler et al. 2022; Liao et al. 2023; Hickey et al. 2024). These graphs consist of conserved genomic regions shared across individuals and divergent sequences representing cultivar- or lineage-specific adaptations (Vaughn et al. 2022; Rice et al. 2023). For plant breeding, pangenome graphs offer several advantages. First, they enable the discovery of causal variants underlying important traits. These include SVs and copy-number changes, which are often missed by short-read or single-reference approaches (Zhao et al. 2021b; Lyu et al. 2023; Zhang et al. 2023). Second, pangenome graphs incorporate a broader set of haplotypes. This improves the accuracy of trait mapping and association studies in polyploids and highly diverse crops (Stein et al. 2017; Hurgobin et al. 2018; Guo et al. 2020). Third, pangenome graphs help identify presence–absence variation in resistance genes and other loci linked to stress adaptation. These traits are often under selection in breeding programs (Bertioli et al. 2019; Schiessl et al. 2019; Vaughn et al. 2022). They thus create a stronger genomic toolkit for marker-assisted breeding and predictive modeling. This can accelerate the improvement of complex traits such as yield, disease resistance, and climate resilience (Henkrar and Udupa 2020; Munyengwa et al. 2021; Samantara et al. 2021; Ayalew et al. 2022; Yang and Ma 2025). As sequencing costs drop and graph-based tools become more efficient, pangenome graphs are becoming an integral part of modern crop breeding strategies (Rice et al. 2023; Hickey et al. 2024).

Peanut (*Arachis hypogaea*; AABB, 2*n* = 4x = 40), also known as groundnut, is a highly nutritious legume rich in protein, fiber, and unsaturated fat (Arya et al. 2016). Although consumed globally, peanuts are a staple in Asia and Africa, and their ready-to-use therapeutic food (RUFT) products have been utilized to eliminate malnutrition in African communities (Guimón and Guimón 2012). As an oilseed cash crop, it also generates revenue and supports the livelihoods of those who grow it (Kelly et al. 1996). However, peanut is a challenging allotetraploid crop for trait mapping due to its genomic complexity (Bertioli et al. 2019). Multi-parent advanced generation intercross (MAGIC) populations provide an effective framework for trait mapping, offering enhanced resolution and genetic diversity (Arrones et al. 2020; Samantara et al. 2021). To rapidly incorporate and map multiple traits of interest in peanut, an 18-parent (16-way) MAGIC peanut population was recently developed, with both LRLP and SRLP sequencing conducted on the population to compare genotyping accuracy (Arrones et al. 2020).

**Fig. 1.**
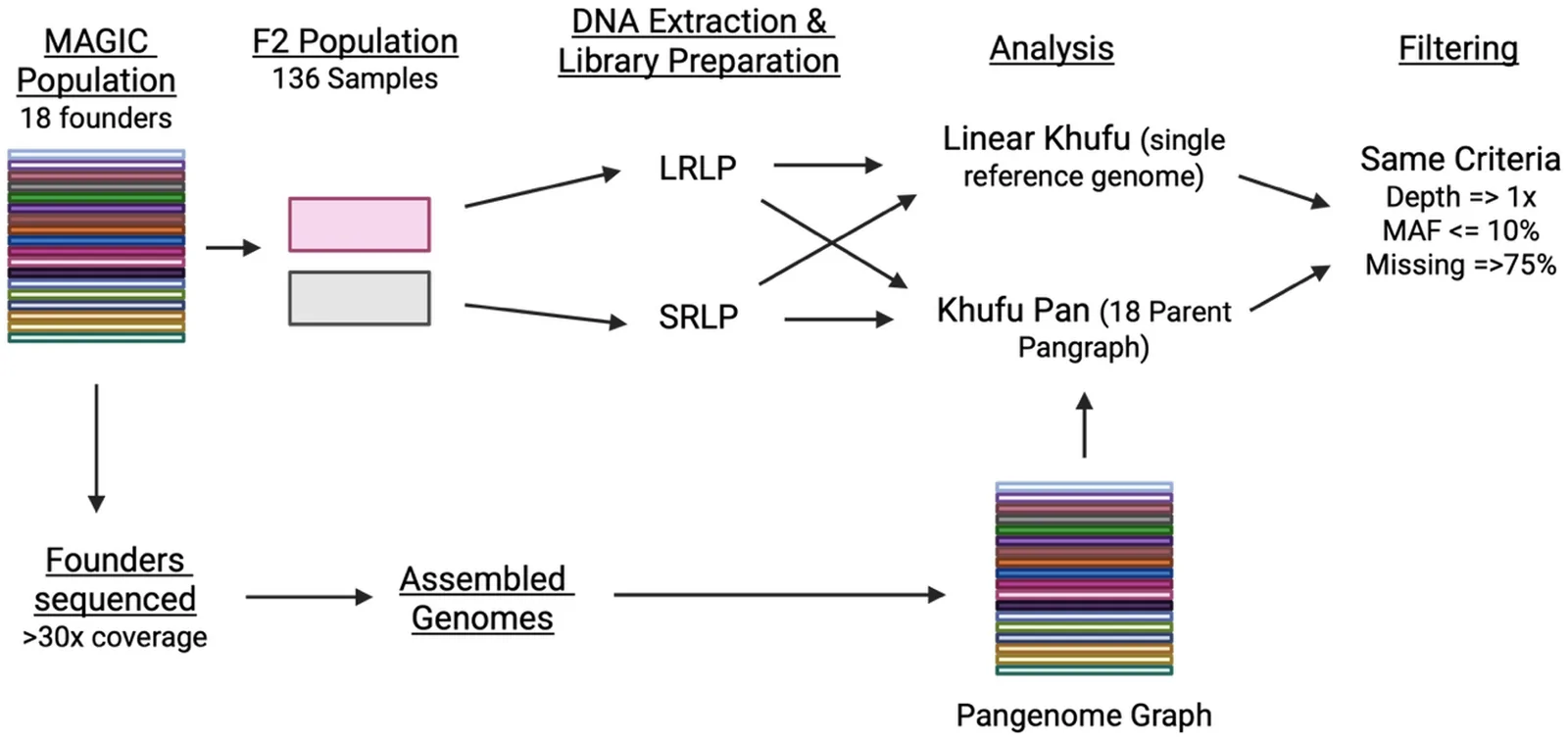
Overview of LRLP and SRLP experimental design and filtering metrics for the MAGIC peanut population. This figure illustrates the workflow and key metrics of the long-read low-pass (LRLP) and short-read low-pass (SRLP) sequencing strategies applied to 136 MAGIC population peanut samples derived from 18 founder lines. Samples were sequenced using PacBio HiFi (LRLP) and Illumina (SRLP) platforms, with nine samples excluded for low yield. Reads were aligned to the *Arachis hypogaea* Tifrunner v2 (TRv2) reference genome as well as an 18-parent pangenome. Genotyping was performed using the Khufu pipeline in two modes: Traditional Khufu, which calls variants from single-reference alignments, and Khufu Pan, which performs variant discovery across the multi-reference pangenome graph to capture lineage-specific and presence–absence variations. Variant calls were filtered based on standardized quality thresholds, including a minimum sequencing depth of ≥1x, minor-allele frequency (MAF) ≤ 10%, and a maximum missing-data threshold of 75%. Filtering was applied uniformly across LRLP and SRLP datasets to ensure comparability between data types. This analytical framework enabled assessment of variant discovery and genotyping accuracy in both linear and graph-based contexts. Created in BioRender. Lee, K. (2026) https://BioRender.com/lfbhv82.

We present the efficacy of the LRLP methodology within a functional peanut breeding framework. Using an 18-parent (16-way) MAGIC peanut population, we demonstrate the development of wet-lab methods and the adaptation of the Khufu analysis pipeline to identify and associate SNPs and SVs with traits of interest (Fig. 1) (Korani et al. 2021; Lou et al. 2021). By aligning both LRLP and SRLP sequences to a single reference and the pangenome graph of select parents, we evaluate the performance and utility of each approach. We demonstrate that LRLP offers a robust, highly accurate alternative to current methods for identifying high-quality genotypes in an active peanut breeding program. Furthermore, we marry the implications of the LRLP methodology to the peanut system to demonstrate its implications in orphan crop breeding.

## Methods

### Population development

The peanut (*Arachis hypogaea*) population analyzed in this study originates from an active breeding program led by the HudsonAlpha Institute for Biotechnology in collaboration with the University of Georgia. The primary objective of the program is to stack desirable, known quantitative trait loci (QTL) from elite parental lines into advanced progeny through strategic hybridization and selection.

Eighteen genetically diverse parental lines (listed in Supplementary Table 1) were initially crossed in pairs. From each pair, seven F_1_ plants were retained following initial crosses performed in January 2019. A half-diallel crossing scheme was implemented in September 2020, using progeny from the initial crosses as parents. In March 2021, 75 four-way crosses were performed using the resulting germplasm. Subsequently, 56 eight-way crosses were made in September 2021 to integrate genetic material from all 18 founders. These lines were then self-pollinated to the F₂ generation for evaluation.

### Whole-genome assembly for MAGIC parents

Eighteen parental lines were traditionally high-coverage sequenced using PacBio HiFi technology for genome assembly. The raw HiFi reads obtained from the sequencer were processed to remove adapters using HiFiAdapterFilt (https://github.com/sheinasim-USDA/HiFiAdapterFilt) (Sim et al. 2022). The filtered reads were assembled using HiFiasm (https://github.com/chhylp123/hifiasm) (Cheng et al. 2021). The “-l0” flag was used to avoid subgenomic chimeras (via haplotype mis-phasing) during the assembly. Assembled genomes were assessed for their quality using the Quality Assessment Tool (QUAST) (https://github.com/ablab/quast) and Benchmarking Universal Single-Copy Orthologs (BUSCO) (https://github.com/metashot/busco), which suggested high completeness and contiguity (Supplementary Table 1). All the assembled genomes, except Bailey, were scaffolded to the reference Tifrunner V2 (TRv2) assembly using Pteranodon, which leveraged the TRv2 assembly (https://www.peanutbase.org/) (https://github.com/w-korani/Pteranodon). Bailey was scaffolded to the Bailey II reference assembly (Newman et al. 2023) rather than TRv2. As a post-assembly check, dotplots were generated against community reference genomes to observe the integrity of the assembled genome using mummerCoordsDotPlotly.R (https://github.com/tpoorten/dotPlotly/blob/master/mummerCoordsDotPlotly.R).

### DNA extraction and library preparation

For long-read sequencing, approximately 100 mg of young leaf tissue was sampled from each of the 136 plants in the HudsonAlpha Institute for Biotechnology greenhouse (Huntsville, AL, USA). Tissue was collected using a hole punch and placed immediately into precooled 2 mL deep-well 96-well Eppendorf plates containing grinding beads. Plates were maintained on dry ice during sample collection and stored overnight at −80 °C prior to extraction.

Tissue homogenization was carried out using a Spex MiniPrep G homogenizer in short bursts, allowing samples to partially refreeze between cycles to preserve DNA integrity. DNA extractions were performed using proprietary PacBio-formulated reagents. Depending on the batch size, either full 96-well plates or 24-tube subsets were processed. Automation was performed on a Thermo Scientific KingFisher Apex magnetic particle processor (catalog #5400930). DNA concentrations were quantified using the Qubit dsDNA High Sensitivity Assay Kit (catalog #Q33230).

To improve read-length distributions, DNA fragments smaller than 10 kb were removed using the PacBio Short-Read Elimination (SRE) Kit (catalog #102-208-300), which typically yielded ∼50% DNA recovery relative to the initial concentration. After SRE cleanup, DNA samples were diluted to ≤10 ng/µL in 300 µL of low TE buffer. Shearing was performed through repeated pipetting cycles using a Hamilton Microlab Prep workstation. Sheared DNA was collected in reduced volumes and further purified with PacBio SMRTbell Cleanup Beads at a 1:1 volume ratio. Library construction was performed using the PacBio HiFi SMRTbell Prep Kit 96 (catalog #103-381-200). Samples were uniquely barcoded for multiplexing, and pooled libraries typically contained 12 samples (ranging from 10 to 13 per pool). Sequencing was performed by SeqCenter (Pittsburgh, PA, USA) using the PacBio Revio system and Revio Flow Cells.

For short-read sequencing, DNA was extracted using the BioEcho EchoLUTION Plant DNA Kit (catalog #010-303-002) in 96-well format. Libraries were prepared using the Twist 96-Plex Library Prep Kit (catalog #10653). Sequencing was carried out on an Illumina NovaSeq 6000 platform at Discovery Life Sciences (Huntsville, AL, USA). To increase data depth and approximate the target coverage of long-read samples, each individual was sequenced in duplicate. Long-read and short-read datasets were deliberately not normalized to identical depths to preserve variability introduced by sequencing yield, thereby avoiding biases from artificial randomization.

### Linear Khufu analysis

Khufu, a proprietary trait-mapping tool originally developed for short-read genomic data (https://www.hudsonalpha.org/khufudata/), was adapted here to accommodate long-read sequencing data (Korani et al. 2021). Khufu, using a single-reference genome (linear khufu), was used for both SRLP and LRLP data. TRv2 serves as the reference genome for both SRLP and LRLP. Linear Khufu analyses consisted of mapping and genotyping, with population-specific filters applied. Mapping is done with bwa (v. 0.7.17). The KhufuVAR module, a subtool of Khufu, was used for variant calling and filtering. Filtering parameters included minimum and maximum depth thresholds set at 1 and 3000, respectively. Additionally, SNPs with a MAF of less than 0.1 or missing in 75% or more of the lines were removed, and lines missing 90% or more of SNP calls were excluded. Khufu automatically filtered out sequences that aligned to multiple loci, and alignment confidence was assessed by comparing the numbers of sequences before and after filtering.

To enhance computational efficiency, KhufuVAR operates in two stages. In Stage 1, individual samples are processed in parallel batches, each aligned to the filtered variant set, and metrics for population-wide MAF, polymorphism, and missing data are computed. In Stage 2, combined metrics are applied across all batches to produce the final filtered dataset. The resulting output is formatted as a Khufu panmap- offering a unified representation of SNPs, short indels, and large indels with positional and allele information.

### KhufuPan methods

KhufuPAN, a graph-based extension of Khufu publicly available at https://github.com/w-korani/KhufuPAN, requires a Graphical Fragment Assembly (gfa) file that incorporates genome assemblies from multiple parents, along with a high-quality, well-assembled reference genome designated as the null reference (Supplementary Fig. 5). This null reference assigns coordinate systems for SNPs and SVs. The pangraph used TRv2 as the null reference and incorporated the genomes from the following cultivars and lines: Bailey, C431, C99R, CC477, CC812, Florida07, GA12Y, GPNCWS17, Georganic, IAC322, ICG1471, Lariat, Marc1, NC94022, NMVal Hap 1, TifNV, CC41, York, and TRv2.

The pipeline starts with a GFA file; Cactus (v2.9.1) was used to generate the pangenome graph using the Cactuspangenome workflow with the following key options: --reference, --outDir, --outName, and all output types set to clip (--vcf clip --giraffe clip --gfa clip --xg clip --haplo clip --gbz clip --chrom-vg clip --odgi clip --chrom-og clip --viz clip --draw clip). Toil (bundled with Cactus) managed execution within a Singularity (v3.11.0) container (Hickey et al. 2024). The process is divided into four major sections (Supplementary Fig. 5): (i) bootstrapping the graph for further processing, (ii) library processing to map and extract filtered variants from each library independently, (iii) combining library subsets into panmap files and filtering for sequencing depth, and (iv) combining all sets into a single panmap file and filtering for population structure.

### Bootstrapping

A GFA file is indexed for Giraffe mapping, GBWT indexes, and GBZ graphs using the vg toolkit (v 1.60.0; command vg autoindex --workflow giraffe -t N) within a Singularity (v 3.11.0) environment. Personalized graph and XG indexes are created with vg convert -x, and snarls are computed with vg snarls. The GFA file, originally generated by Cactus (v 2.9.1; cactus-pangenome --reference … --gfa clip --xg clip --gbz clip --haplo clip), is then deconstructed using vg deconstruct -a -t N to generate a parental VCF file (the calls of linear genomes that constructed the graph). The VCF file is filtered through multiple steps to create the bootstrapping dataset, which contains highly confident calls that segregate across the linear genomes, excluding the null reference genome (the genome used as a positional reference for mapping). In addition, it defines and indexes the unique alleles for each variant.

### Library processing

This section is run for every sample independently. This process may be run using Khufu core standards (Korani et al. 2021; Liao et al. 2023) or by mapping fastq files for single- or paired-end reads directly to the GFA graph to achieve the same outcome (Sirén and Paten 2022; Wright et al. 2025). In KhufuPAN, this corresponds to the sample-mapping script, which uses VG Giraffe (v 1.60.0; vg giraffe -p -t N -Z graph.gbz -f R1.fastq -f R2.fastq) for alignment. Personalized subgraphs are optionally generated when the parameter --kmer > 0, invoking KMC (v 3.x) to derive sample-specific k-mers and VG haplotypes to construct the individualized graph (vg haplotypes -k sample.kff -g sample.gbz). VG filter (--min-mapq 60) screens low-quality mappings, followed by vg pack and vg call (-a -t N -g graph.gbwt -r snarls) to produce sample-specific VCF files. In either case, VG Giraffe is used for mapping, and VG call is used to generate sample VCF files. The calls are filtered and adjusted to the variants/alleles and indexes of the bootstrapping dataset. Variants and mapping reads were filtered at multiple stages to retain only high-confidence sites. During graph bootstrapping, variants were required to have a Phred-scaled quality score of ≥60 and to occur in more than one parental genome; loci containing ambiguous (N/X) bases were removed. During sample mapping, reads with mapping quality <60 or base quality <5 were excluded (vg filter --min-mapq 60; vg pack -Q 5). Genotypes with sequencing depth less than 1x were masked.

At the population level, only variants with a MAF ≥0.1 and <75% missing data across samples were retained, and monomorphic or null-reference-only variants were discarded. Variants present in the pangenome graph but lacking sufficient evidence in a sample were treated as missing data rather than confirmed alleles.

### Combining a subset

To shorten processing time and reduce computational resources, samples are divided into two sections: this section and the next. In this section, a subset of samples (∼100) is filtered for sequencing depth, and calls are collected from the bootstrapping dataset. Filtering thresholds (--min depth = 1; --max depth = 50) and merging are performed by the combination script, implemented in Bash with standard awk, sort, and paste operations. A panmap file is created for each subset that contains the same set of variants/alleles as the bootstrapping dataset.

The population may not segregate for all bootstrapping dataset variants. In addition, not all variants are reasonably covered across all samples due to the nature of skim-seq. Therefore, KhufuEnv (https://github.com/w-korani/KhufuEnv) is used to combine all subsets (panmap files) into a single population panmap file and to apply filtering, eg for missing data, low MAF, and polymorphic markers (Wright et al. 2025).

#### Validation of 1x genotyping accuracy

To assess the accuracy of low-coverage variant genotyping, the 18 pangenome founder accessions (originally sequenced at high depth) were used as a ground truth set and evaluated independently under both the pangenome and linear-reference pipelines. To simulate LRLP sequencing conditions, raw HiFi reads for each founder were first fragmented into non-overlapping 7,000-bp k-mers using fastq2kmer (KhufuEnv v2; kmer size = 7,000, window = 7,000), matching the read-length profile of LRLP libraries. The resulting k-merized reads were then subsampled to 1x genome coverage (target genome size = 2,672,191,898 bp) using fastqSubSampling (KhufuEnv v2).

For pangenome validation, variants were called independently for both the 1x subsampled and full-depth datasets by aligning to the pangenome graph (gfa) and calling variants using KhufuPAN, followed by site-level filtering (site missingness ≤0.80, MAF ≥0.10, no sample-level missingness filter applied). Prior to comparison, allele sequence identity between the two filtered datasets was confirmed by cross-referencing the corresponding per-site allele FASTA files; all 459,787 allele entries shared between the two filtered datasets were sequence-identical, confirming that allele indices are consistent across depth conditions.

For linear-reference validation, the same 1x subsampled and full-depth reads for founders were aligned to the TRv2 reference genome using pbmm2 and variants genotyped using the Khufu linear pipeline, with identical site-level filtering (site missingness ≤1.0, MAF ≥0.10). Genotype calls were output in hapmap format with missing data encoded as “-,” homozygous calls as a single nucleotide, and heterozygous calls as a two-character alphabetically ordered string (eg “AG”).

Callability was defined per sample as the fraction of full-depth-filtered sites (with a non-missing call for that sample) that also carried a non-missing call in the 1x data, restricted to sites present in both filtered datasets. Concordance was defined as the fraction of co-called sites where the allele assignment agreed exactly. Discordant calls were further classified as (i) allele dropout, where the full-depth call was heterozygous and the 1x call was homozygous; (ii) overcall, where the full-depth call was homozygous and the 1x call was heterozygous; (iii) wrong allele, where both calls were homozygous but for different alleles; and (iv) het mismatch, where both calls were heterozygous but differed in allele combination. Allele–identity accuracy was calculated as the fraction of co-called sites that were concordant or differed only in zygosity (ie excluding dropout and overcall from the denominator). All analyses were performed in Python 3 using pandas (scripts available at github.com/Kendall-Lee/Scripts/pangenome). Per-sample results are reported in Supplementary Table 2.

#### Other analysis

Long-read low-pass and SRLP datasets were aligned to the TRv2 reference genome using minimap2 (v2.26) and bwa (v0.7.17), respectively. Whole-genome coverage metrics were calculated from the resulting BAM files using bedtools (v2.28.0). Gene–space coverage was assessed by intersecting the alignment files with the TRv2 gene annotation using the bedtools intersect function. Of the 136 plants sampled, six failed to produce sufficient sequencing data for inclusion in downstream analyses. Three additional samples were identified as technical duplicates and consolidated with their corresponding primary entries, yielding 127 unique LRLP libraries. Of these, three lacked a corresponding short-read (SRLP) library and were excluded from paired comparisons, leaving 124 complete LRLP–SRLP pairs used for whole-genome coverage and depth analyses (df = 123). For confidence in the alignment analyses, four duplicate entries in the source data lacked a corresponding SRLP value and were excluded after data reshaping, yielding 126 complete pairs (df = 125). Gene–space coverage was assessed for 120 of the 124 paired samples; four samples (W.2024.SP.CA.D7.F1.03, W.2024.SP.CW.D3.F1.24, W.2024.SP.HCCA.D1.F1.03, and W.2024.SP.RB.D3.F1.09) were excluded due to insufficient LRLP sequencing depth (0.22–0.35x), which yielded filtered gene–space coverage values (8–21%) inconsistent with the remainder of the dataset (df = 119). All degrees of freedom reported in the manuscript reflect the number of complete paired observations available for each specific analysis and are the values returned directly by R's t.test (paired = TRUE) function.

Structural variations in the linear analysis were identified using PacBio's pbmm2 and pbsv (v. 2.9.0) pipelines, with TRv2 as the reference genome. The functional effects of these SVs were assessed using snpEff (Cingolani et al. 2012). Analyses included both merged and individual VCF files. Correlation and paired t-test analyses were performed in RStudio (v. 2023.06.1+524) using the stats (v. 4.3.1) package. All correlations employed the Pearson method via the cor.test function.

The TSWV resistance locus is a copy-number variant of an NLR-class disease resistance gene cluster and is not recoverable by breakpoint-based SV callers, which require split reads spanning an insertion boundary. To genotype the locus, we extracted the full insertion sequence from the NC94022 parental line, which carries the insertion, and extended it with 500 kb of flanking genomic sequence on either side to provide sufficient alignment context. This extended reference segment was used as the mapping template for both LRLP and SRLP datasets, with reads aligned using minimap2 (v2.26) and bwa (v0.7.17), respectively, under standard parameters. Coverage across the insertion region was extracted from the resulting BAM files using samtools depth via a custom script (available at https://github.com/Kendall-Lee/Scripts/blob/main/sv-cnv/TSWV_insertion_genotype.sh), and the percentage of insertion bases with ≥1x coverage (Insertion_Pct_Covered) was calculated as the primary genotyping metric. Individuals were classified as PRESENT (>15% of the 63.9 kb insertion covered with internal read), ABSENT (0%), or UNCERTAIN (0.1–9.9%); the 15% threshold corresponds to the minimum coverage expected from a single long read spanning the insertion interior and was validated by manual inspection of alignments in IGV across both data types. Per-sample coverage metrics and genotype calls are provided in the supplemental data at https://doi.org/10.6084/m9.figshare.32374161.

An interactive heatmap of variant density was developed using a custom-made R Shiny application (Supplementary Fig. 8). The output VCF file from the minigraph cactus software was used. The variants were parsed from the VCF files using the VariantAnnotation package, and genomic windows were generated using the GenomicRanges package, both in R. Each chromosome was partitioned into non-overlapping 10 kb bins, and variants per genome per bin were counted. Hypervariable regions were identified as having more than 400 variants per 10-kb window and were excluded from analysis because they are likely forced alignments. The graph was created using the ggplot2 package in R, with the *Y*-axis representing chromosome position (Mb) and the *X*-axis representing genomes/samples. The color intensity represents the density of the variants per kb, as shown in the legend. This application allows users to select the chromosomes and genomes of interest, dynamically generating a heatmap with genomic regions on the Y-axis, genomes on the *X*-axis, and color intensity representing variant density (variants/kb). The application has been released here: https://sameerpokhrel.shinyapps.io/ShinyAppMAGICPopulation/.

#### Use of large language models

Claude (Anthropic, model claude-sonnet-4–6, accessed via claude.ai) was used to assist with three tasks: (i) drafting and troubleshooting portions of the analysis scripts used for coverage, depth, and variant-calling comparisons; (ii) generating and refining plotting scripts (ggplot2/R) used to produce figures from processed data tables; and (iii) proofreading manuscript text for clarity and grammar. In each case, the authors supplied the underlying data, analysis logic, or draft text and iteratively reviewed and edited the model's output; no results, figures, or conclusions were generated autonomously by the model. The authors take full responsibility for the accuracy of all content produced with this assistance and confirm the absence of plagiarism.

## Results

### LRLP yield quality sequencing statistics

We sequenced individuals from an active peanut breeding population to show the applicability of LRLP. These individuals are from 16-way hybrids that were selfed after crossing. The population segregates for known QTL that can be selected for using genomic information. Given the pedigree and early-generation advancement, many genotypes in our sample are expected to be heterozygous. Peanut is an allotetraploid crop (AABB; 2n = 4x = 40) comprising two subgenomes, A and B, each consisting of 10 chromosomes. The reference genome used in this study (Tifrunner v. 2) represents one complete set of A- and B-derived chromosomes. Consequently, a mean sequencing depth of ∼1x corresponds to approximately one read per site across each subgenome. At about 1x depth of coverage, sequencing will rarely determine the genotypic state but will instead reveal only the allelic state of one haplotype.

Sequencing statistics are reported from PacBio's SMRTLink software. These statistics are important to understand the efficacy of our sample preparation pipeline. Pools consisted of 10–13 samples each, with the majority containing 12. Overall yield per sample, base quality score, mean read length, and median read length were evaluated to ensure high-quality sequences were being produced. Of 136 samples, nine were removed due to low sequencing yield. An average of 4.25 Gb of HiFi sequence data was generated per sample, and 98.2% of HiFi base pairs across all samples had a Phred score above 20, indicating very high data quality (Table 1). Mean read length varied between 4,151 bp and 13,169 bp, with an average of 7,927 bp (Table 1, Supplementary Fig. 1). Median read length had a similar range of 2,278–13,185 bp with an average of 7,046 bp (Table 1). Short-read low-pass sequencing statistics were as expected, with an average yield of 4.2 Gb of data per sample and an average sequence length of 137 bp. All sequences passed the mean quality threshold of Q20.

**Table 1.** Raw sequencing statistics of long-read low-pass data of 127 MAGIC population peanut lines.

Total reads	Average Gb	bps ≥ Q20	% bp ≥ Q20	Mean read length (bp)	Median read length (bp)
553,955	4.25	4,170,816,749	98.21	7,927	7,046

### Genome coverage and site depth are substantially improved by LRLP

**Fig. 2.**
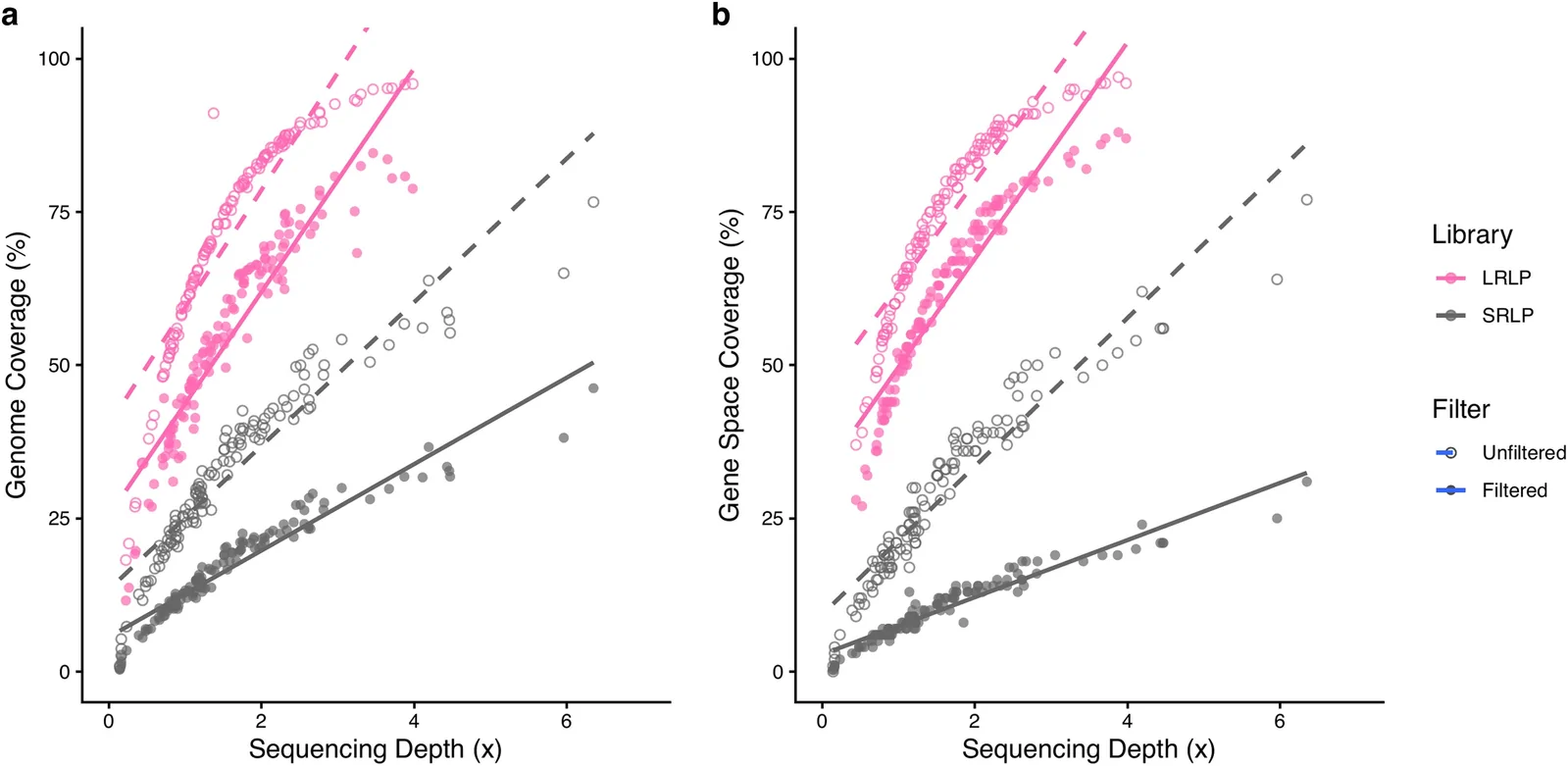
Whole-genome and gene–space read coverage vs sequencing depth in TRv2. Reads were aligned to the *A. hypogaea* Tifrunner reference genome (TRv2) to assess sequencing depth and coverage across 124 paired LRLP–SRLP samples. a) Whole-genome read coverage plotted against sequencing depth for both long-read (LRLP) and short-read (SRLP) libraries before and after filtering for potential misalignments. Average sequencing depth did not differ significantly between LRLP and SRLP datasets (t(123) = 0.223, *P* = 0.824), with mean depths of 1.63x and 1.68x, respectively. However, whole-genome coverage differed substantially: unfiltered LRLP datasets showed 71.29% coverage, reduced to 55% after filtering, whereas SRLP datasets showed 32.67% coverage unfiltered and 17.3% after filtering (unfiltered: t(123) = 19.68, *P* < 2.2 × 10^–16^; filtered: t(123) = 23.83, *P* < 2.2 × 10^–16^). b) Gene–space coverage plotted against sequencing depth before and after filtering for potential misalignments, assessed across 120 paired samples. LRLP libraries achieved an average filtered gene–space coverage of 57.9%, slightly exceeding overall filtered genome-wide coverage, while SRLP libraries averaged only 11% filtered gene–space coverage (unfiltered: t(119) = −24.30, *P* < 2.2 × 10^–16^; filtered: t(119) = −35.72, *P* < 2.2 × 10^–16^).

Reads were aligned to the single-reference genome of Tifrunner version 2 (TRv2) to assess depth and coverage (Bertioli et al. 2019). Depth (coverage depth) is the average number of times a position in the genome has been sequenced, whereas coverage is the percentage of the entire genome that has been sequenced at least once. Both metrics were calculated for each sample. A paired t-test shows that there is no significant difference between the long- and short-read sequence depths (t(123) = −0.22, *P* = 0.82), and the average difference in depth across samples is 0.028x (Fig. 2a). The average depth for long reads was 1.63x, and the average unfiltered whole-genome coverage was 71.29%, whereas for short reads, it was 1.68x depth and 32.67% (Fig. 2a, Supplementary Fig. 2). After filtering for highly probable misalignments, long-read samples had an average coverage of 55%, and short-read samples had an average coverage of 17.3%. While the difference in depth between short and long reads was not statistically significant, both unfiltered and filtered coverage differed significantly between read types (unfiltered: t(123) = 19.68, *P* < 2.2×10^–16^; filtered: t(123) = 23.83, *P* < 2.2×10^–16^) (Fig. 2a). The gene–space (the portion of the genome containing genes) covered was also analyzed using the gene annotation for the TRv2 (Bertioli et al. 2019). The average gene–space coverage for long reads after filtering was 57.9%, slightly higher than the overall filtered coverage (Fig. 2b). This is likely because genic regions exhibit greater sequence complexity. In contrast, after filtering, short reads cover only 11% of the gene–space on average and are significantly lower than long reads (unfiltered: t(119) = 24.296, *P* < 2.2×10^–16^; filtered: t(119) = 35.721, *P* < 2.2×10^–16^) (Supplementary Fig. 3).

### LRLP improves confidence in accurate alignment by reducing the number of discarded multiple alignments

One of the key advantages of long-read sequencing over short-read sequencing is its higher likelihood of uniquely aligning to the reference genome, resulting in fewer sequences being discarded due to multiple alignments. We refer to the percentage of reads retained after filtering for misalignment probability as the alignment confidence, ie the proportion of reads that align to a single location in the reference genome. Misalignment filtering is applied when a read maps to multiple regions of the genome. On average, long reads had a confidence of alignment of 92.9%, significantly higher than the 54.9% confidence of alignment observed for short reads (t(125) = 142.51, *P* < 2.2×10^–16^) (Supplementary Fig. 4). This increased alignment confidence in long reads enables more comprehensive genome coverage, especially in repetitive or complex regions that are challenging for short reads to resolve.

### Pangraph analysis captures more SNP, indel, and SV calls than using a single reference

#### Pangraph construction

To maximize the efficiency of profiling genotype information from long-read sequencing data, we developed a customized pipeline named KhufuPAN (available at https://github.com/w-korani/KhufuPAN) (Supplementary Fig. 5). This pipeline integrates the powerful genome graph construction tool Cactus (Hickey et al. 2024) with variant-aware graph alignment and variant calling via VG Giraffe v. 1.60.0 (Garrison et al. 2018; Sirén and Paten 2022), all wrapped in custom scripting modules designed to handle large datasets efficiently. KhufuPAN accepts both short-read and long-read data in fastq format and outputs high-confidence, filtered variant calls. After construction of the pangenome graph- which incorporates 18 parental peanut genomes with TRv2 as the anchor reference (Bertioli et al. 2019) (Supplementary Table 1), the pipeline proceeds through three main stages: calling, combining, and filtering alleles (Supplementary Fig. 5). Reads are aligned to the graph using Giraffe, following preprocessing steps that ensure high-quality variant anchors. Alleles are mapped back to this filtered set, and metrics such as missing-data frequency and minor-allele frequency (MAF) are computed to enable flexible downstream filtering. A site was considered captured if a genotype call (any allele) was present at a genomic coordinate corresponding to a variant in the high-confidence bootstrapping dataset (filtered pangenome variant set). Finally, a novel output format called a panmap is generated (Wright et al. 2025). Panmap files store essential information for each variant type (eg chromosome, position, and allele length), graph-based allele assignments, and genotype calls in a numeric matrix format. A corresponding fasta file provides allele sequences for each variant. The panmap format can be efficiently converted to standard formats such as Variant Call Format (VCF) or Hapmap to support compatibility with external analysis and breeding software.

In terms of computational performance, KhufuPAN required, on average, a maximum of 47.9 GB of RAM and 24.15 CPU hours per sample, with most steps supporting multithreading, making it scalable for high-throughput breeding applications.

**Fig. 3.**
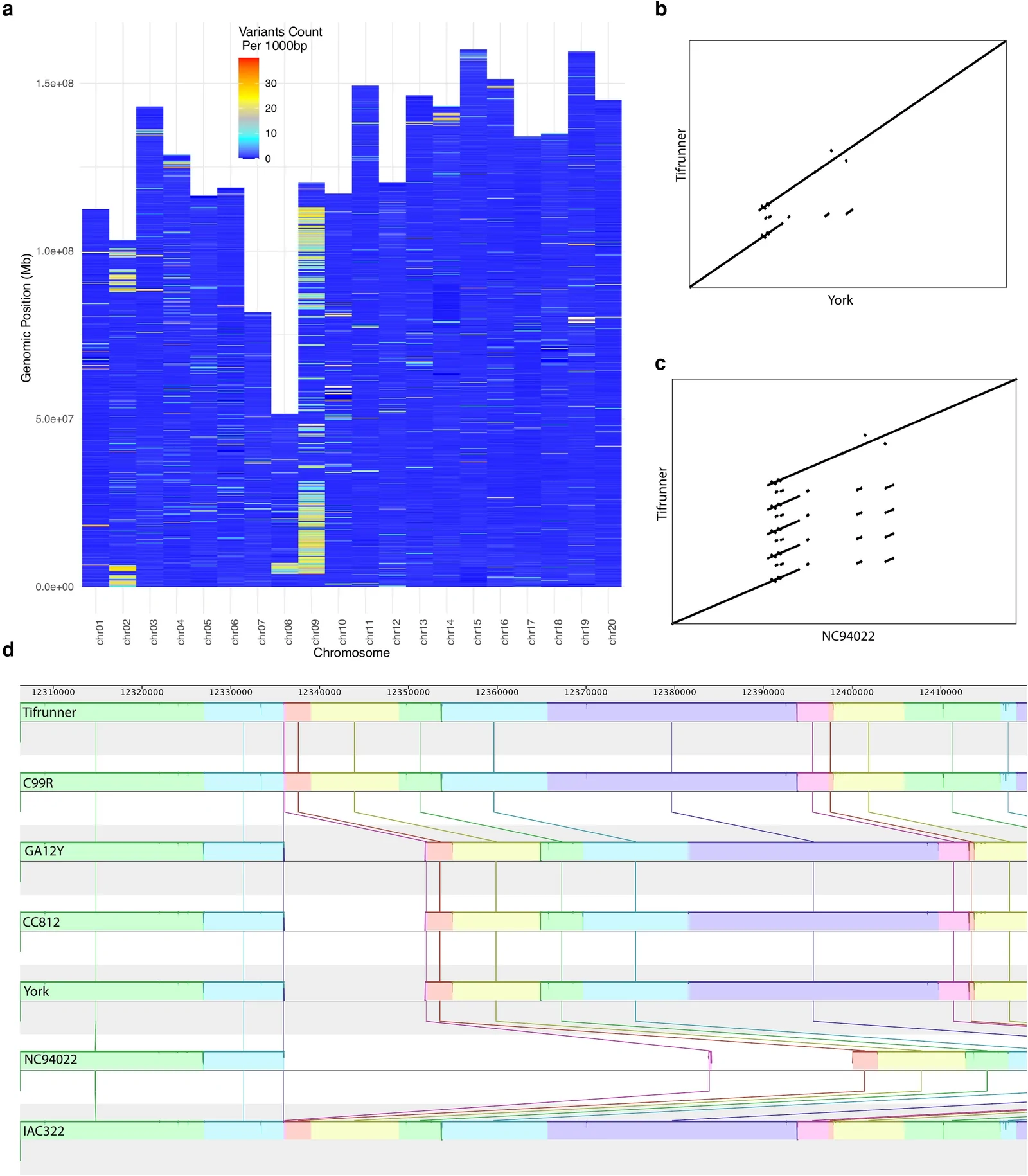
Panels a–d collectively characterize pangenomic variation in the 18-parent MAGIC population, with b–d focusing on the TSWV resistance locus as a case study. a) Heatmap of pangenomic variation across all 18 newly sequenced cultivars and the TRv2 reference. The genome was divided into windows of 100 kb with 10 kb overlap. Counts were transformed to a per-kb scale for ease of interpretation. Manual inspection indicated that 811 (0.3% of total) hypervariable windows (>40 variants per 1 kb) were forced alignments; these were removed from the heatmap. b) Dotplot of orthologous genomic region (Tifrunner Chr.01: 12306000–12426000) associated with minor TSWV resistance in York. c) Dotplot of the same region associated with full TSWV resistance in NC94022. d) Panmap alignments of the TSWV region across multiple cultivars. Colors indicate alignment blocks, and lines indicate orthology. Large, unaligned sequences are indicated by unblocked horizontal spans, illustrating that tandem duplications (evident in dotplots) form a linearized allelic series in the panmap.

The pangenome graph constructed using the KhufuPan pipeline was visualized to assess total variation and to check known regions of interest for genome alignment validation (Fig. 3; Supplementary Fig. 5). Regions of forced alignment were removed. The founder pangraph called 4,443,991 potential variant sites.

#### Pangraph alignment

**Fig. 4.**
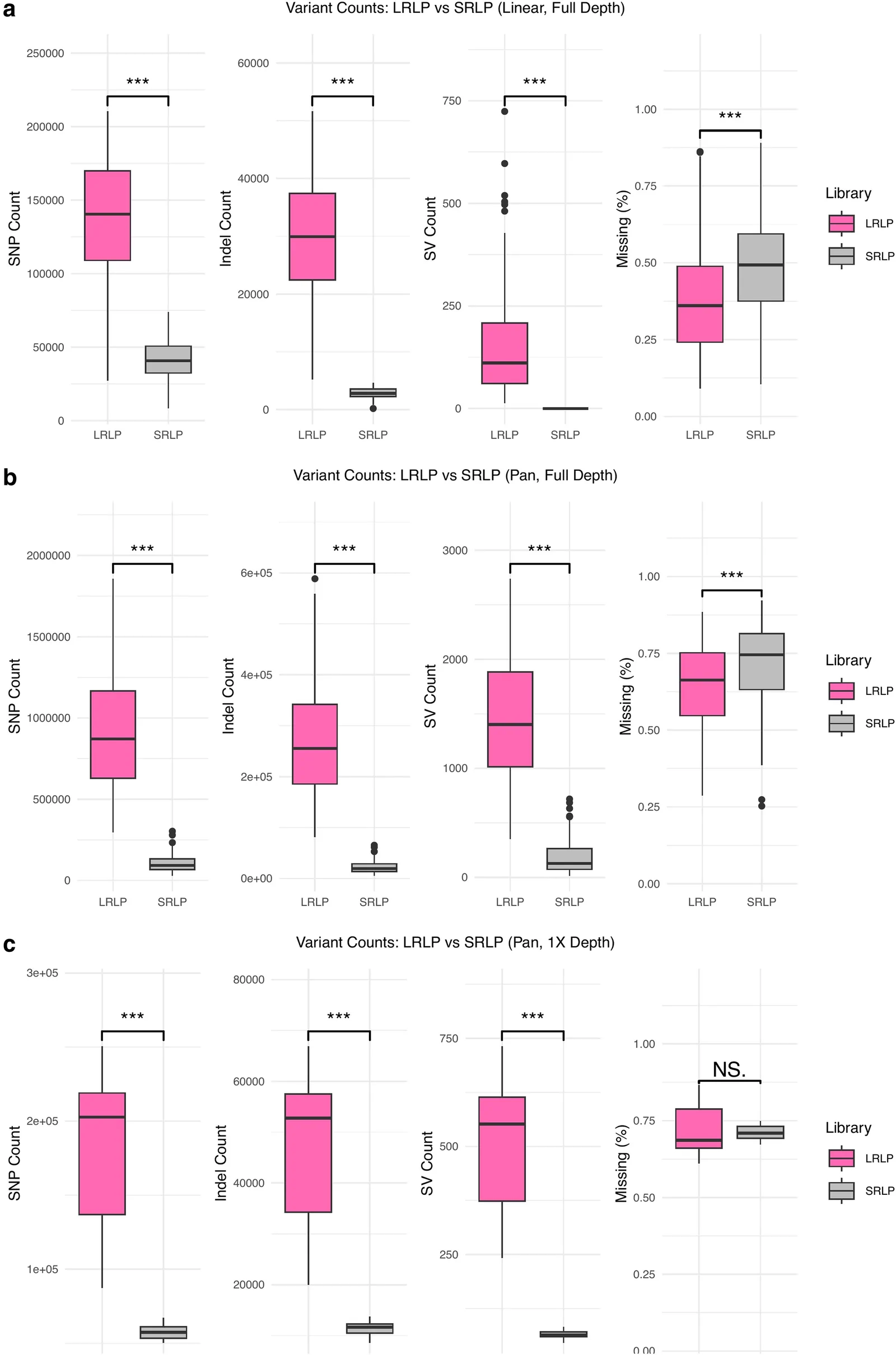
Comparison of variant discovery between long-read (LRLP) and short-read (SRLP) libraries across linear and pangenome analyses. Variants were called using Khufu against the Tifrunner reference genome (TRv2) and the pangraph across different sequencing depths and coverages. a) Under the linear-reference framework at full depth, LRLP libraries identified significantly more SNPs, indels, and SVs compared to SRLP libraries, while SRLP libraries exhibited a significantly higher percentage of missing data (W = 4808, P < 0.001). On average, LRLPs identified 138,754 SNPs, 30,032 indels, and 150 SVs per line, while SRLPs identified 41,745 SNPs, 2,876 indels, and 0 SVs per line. b) Using the pangraph at full depth, LRLP libraries again detected substantially more variants (3,272,323 total) compared to SRLP (482,960 total), including a marked increase in indels and SVs. SRLP libraries also exhibited significantly higher missing data rates than LRLP (W = 5502, P < 0.001). On average, LRLPs identified 985,435 SNPs, 260,382 indels, and 1,445 SVs per line, while SRLPs identified 169,987 SNPs, 27,306 indels, and 527 SVs per line. c) At normalized coverage of 1x depth in the pangraph, LRLP libraries still identified significantly more SNPs, indels, and SVs per sample than SRLP. Missing data did not differ significantly between read types (W = 1347, P = 0.065), with LRLP averaging 71.5% and SRLP averaging 63.7%. Across LRLP samples, an average of 533,759 SNPs, 129,805 indels, and 831 SVs were called, while SRLP samples averaged 100,375 SNPs, 14,645 indels, and 238 SVs. Together, these results highlight the greater power of long-read datasets to capture genomic variation, particularly complex variants, compared to short-read datasets.

The 18-parent pangraph captured 3,271,583 alleles across the variant sites on the filtered pangenome variant set for LRLP reads, while SRLP captured 426,608 alleles across the variant sites when full-depth coverage was used on the pangraph (Fig. 4, Supplementary Fig. 5). Genotypes were considered only when a physical read covered the site. While there was no significant difference between the percentage of missing data for SRLP reads and LRLP on the pangraph at full depth, LRLP sequences identified significantly more variation (Fig. 4b). On average, SRLP identified 169,987 SNPs, 27,306 insertions/deletions (indels), and 527 large SVs per sample, while long reads found 985,435 SNPs, 260,382 indels (2–1000 bp), and 1,445 large SVs (>1000 bp) per sample (Fig. 4b). To normalize for depth, we downsampled LRLP and SRLP samples that had >1x depth to 1x depth and called alleles utilizing the pangraph. At the adjusted depth, LRLP data called 2,275,851 alleles across the filtered pangenome variant set on the pangraph, and SRLP data called 297,192 alleles across the filtered pangenome variant set. On average across LRLP samples, 533,759 SNPs, 129,805 indels, and 831 SVs were called, and in SRLP samples, 100,375 SNPs, 14,645 indels, and 238 SVs were called (Fig. 4c). Unlike for the linear dataset or pan dataset with full coverage, the percentage of missing data was significantly higher in the 1x depth LRLP data with an average of 71.5% missing than 1x depth SRLP data, which has an average missing percentage of 63.7% (Fig. 4). Despite a higher percentage of missing data in the normalized reads, LRLP shows significantly higher allele capture across all metrics. This highlights the wealth of information that LRLP sequences offer, compared to short reads, when combined with the power of pangenomics. SNP density across the genome was largely uniform for both LRLP and SRLP when mapped to the linear reference, with predictable low-density regions on Chr.05 and Chr.15 corresponding to pericentromeric regions (Supplementary Fig. 6). In contrast, SRLP panmap alignments exhibited extensive windows with zero or near-zero SNP calls across nearly every chromosome—most severely on Chr.02 and Chr.09, where 45 and 48 consecutive 1 Mb windows, respectively, contained no calls. Long-read low-pass panmap alignments showed far fewer such gaps under identical filtering parameters, consistent with long reads’ ability to traverse complex and repetitive regions of the pangenome graph that short reads cannot resolve.

#### Linear analysis

Against a single-reference genome, the samples sequenced with LRLP analyzed with Khufu called a total of 235,583 SNPs across all samples after applying several filters: quality control, uniquely mapping reads, >1x depth, MAF threshold (SNPs with a value lower than 10% are dropped), and removing missing data across SNPs (SNPs with calls missing from 75% or more of the lines). The samples sequenced with SRLP data were analyzed using Khufu, which called 35% of the LRLP calls across all samples under the same filtering parameters (Fig. 4a, Supplementary Fig. 7). The drastic increase in alleles increases the likelihood of correctly correlating an SNP to a trait of interest. With a single-reference genome, Khufu can also capture small indels. Across all samples under the same filtering parameters as for SNPs, LRLP sequences had over 74,814 indels called with an average length of 10.84 bp, and SRLP sequences had 6,404 sites where an indel was called with an average length of 4.62 bp. Only 3,498 indel sites were shared between the LRLP and SRLP data (Supplementary Fig. 7). Genome-wide, SNP density in the linear hapmaps was evenly distributed across all 20 chromosomes with no large call deserts outside of pericentromeric regions, confirming that low SNP counts in individual regions reflect coverage gaps in the genome structure (Supplementary Fig. 6).

#### Validation of 1x genotyping accuracy using full-depth founder truth set

To evaluate the accuracy of low-coverage (1x) variant genotyping, we used the pangenome founder accessions as a ground truth set and assessed performance independently for both the pangenome-based and linear-reference pipelines (Supplementary Table 2).

For pangenome genotyping, founder reads were downsampled to 1x, and variants were called using the same graph-based pipeline applied to the experimental population. After quality filtering (site missingness ≤80%, MAF ≥10%), 249,805 variant sites were retained in the 1x dataset and 871,531 in the full-depth dataset. Among the 224,272 sites shared between filtered sets, per-sample callability at 1x ranged from 17.1 to 37.8% (mean 31.2%), reflecting the expected increase in missing data at low coverage. Among co-called sites, overall concordance was 70.1% (range: 42.5–83.1%). The dominant source of discordance was allele dropout, aka heterozygous full-depth calls reduced to homozygous at 1x due to stochastic single-allele sampling. This accounted for 24.0% of co-called sites. Excluding zygosity errors, wrong-allele errors occurred at 6.3% of non-dropout co-called sites, yielding an allele–identity accuracy of 92.2%.

For comparison, the same scheme was analyzed against a linear reference at both depths under identical conditions. Linear genotyping achieved higher callability (mean 50.3%) and concordance (87.1%), with substantially lower dropout (11.8%) and wrong-allele rates (0.7%), yielding an allele–identity accuracy of 98.7%. This performance advantage is expected: biallelic SNP calling against a fixed reference is a fundamentally simpler task than multi-allele pangenome haplotype resolution and requires less per-site coverage to produce a confident call. However, the linear pipeline cannot detect structural variants, multi-allelic sites, and novel sequences that motivate the pangenome approach; the two analyses therefore address complementary aspects of genetic variation.

In both pipelines, two founders, NMValA and CC477, showed substantially lower concordance (pangenome: 42.5 and 54.6%; linear: 44.5 and 59.6%), driven by elevated dropout rates (pangenome: 48.1 and 39.9%; linear: 50.3 and 38.2%). The consistent pattern across independent pipelines confirms that these outliers reflect elevated background heterozygosity in these accessions rather than pipeline artifacts. Together, these results demonstrate that 1x pangenome genotyping reliably identifies the correct allele when a call is made and that the primary source of error is coverage-driven zygosity loss rather than allele misidentification.

#### Structural variation and impact

Large SVs are often missed by short-read mapping because most short reads are only 150 base pairs (bp) in length and typically ∼137 bp after adapter trimming—a length that is insufficient to span many SVs (Mahmoud et al. 2019; Wenger et al. 2019). In contrast, LRLP sequencing overcomes this limitation by frequently spanning structural rearrangements and repetitive regions, enabling more complete SV detection.

We initially called SVs in our peanut breeding lines using the pbsv pipeline from PacBio, aligning sequences to the TRv2 reference genome (Bertioli et al. 2019). Across all lines, a total of 107,178 unique SVs relative to the reference genome were identified, with an average of ∼150 SVs being found per sample (Fig. 4a). Insertions and deletions represented the most prevalent SV types. The average insertion length was 173.74 bp, and the average deletion length was 1,246.35 bp, both exceeding the typical length of short reads and further highlighting the advantage of long-read sequencing in SV discovery.

To explore the potential biological consequences of these SVs, we used the SnpEff software to annotate and predict effect classes (Cingolani et al. 2012). SnpEff classifies variant impacts into four categories—high, moderate, low, and modifier—based on their predicted disruption to the coding sequence or gene structure. To assess the overall impact at the population level, VCFs from all samples were merged, and unique variants were evaluated. In total, 1,221 high-impact, 464 moderate-impact, 252 low-impact, and 96,767 modifier-impact SVs were detected among all lines (Supplementary Table 3). Of the 1,221 high-impact variants, the majority were deletions (843), followed by insertions (304), duplications (110), inversions (14), and breakpoints (2).

These results demonstrate that LRLP sequencing not only enables the identification of variants missed by traditional short-read approaches but also uncovers high-impact genomic changes potentially relevant to trait expression and selection in breeding populations.

### LRLP identifies causal SV in peanut

To assess whether LRLP reads improve the detection of large structural insertions missed by short-read sequencing, we examined the genomic region containing the tomato spotted wilt virus (TSWV) resistance-associated insertion (Chr 01: 12456955-12520822 region in *Arachis hypogaea* cultivar NC94022) (Fig. 3a) (Thompson et al. 2025; Bowen et al. 2026). Visual inspection in IGV revealed that LRLP has the ability to capture continuous read coverage across the complete insertion locus in samples that have the insertion, whereas SRLP data failed to align within the inserted segment (Supplementary Fig. 9). This example illustrates that long-read low-pass sequencing can recover known, biologically and agronomically important structural variants that are undetectable with short reads. However, this locus was selected as a benchmark because of its presence in the founder. NC94022 is well documented; the same coverage-based approach used in KhufuPAN can be used to detect novel structural variants from low-pass long-read data. Using this coverage-based approach, we identify 16 samples in the population that have the TSWV resistance insertion with LRLP. The identification of these SV-containing lines now enables targeted selection of TSWV-resistant genotypes in subsequent breeding generations, demonstrating the direct utility of long-read sequencing for cultivar improvement.

## Discussion

### Scalability and applications of LRLP sequencing

Long-read sequencing is increasingly recognized across multiple scientific domains for its superior ability to capture complex genetic variation, especially SVs compared to conventional short-read methods (Merker et al. 2018; Mahmoud et al. 2019; Malmberg et al. 2019; Wenger et al. 2019; Fujimoto et al. 2021; Zhao et al. 2021b). Despite these added benefits, LRLP sequencing has seen limited adoption due to barriers in high-throughput sample preparation. One significant constraint is the extraction of HMW DNA for long-read sequencing, which is traditionally performed with a low sample volume.

In contrast, short-read sequencing continues to dominate large-scale genotyping efforts due to its compatibility with automated workflows and lower cost. However, the limited resolution of SRLP impedes the detection of key genetic features, particularly SVs, and its shorter read lengths contribute to frequent misalignments and data loss during filtering processes (Mahmoud et al. 2019). Orphan crops generally have fewer genomic resources than major crops, and few have been used for long-read sequencing for genotyping. Although short reads are valuable for SNP genotyping, long-read sequencing can provide a more comprehensive view of genome structure, particularly in diverse and repetitive regions, thereby accelerating marker–trait associations for orphan crops.

Our optimized high-throughput protocol for HMW DNA extraction and library preparation yields medium-quality DNA at lower input concentrations, while enhanced post-extraction cleanup steps, such as bead-based purification and size selection, help maintain data quality. Samples were pooled for sequencing, targeting a depth of 2–3x. The LRLP workflow yielded an average sequencing depth of 1.63x (range: 0.5x–4x). Long-read low-pass provided more uniform coverage and greater site depth across the genome than SRLP. These improvements likely reflect the inherently higher read-mapping quality, which results in fewer multimapped reads. In contrast, PCR-amplified short-read libraries can show regional coverage bias because certain fragments are over- or underrepresented during library preparation.

Previous studies on low-depth long-read sequencing were mostly limited to small-scale implementations. For example, Malmberg et al. (2019) used Oxford Nanopore's MinION to sequence nine *Brassica napus* lines at 2x–5x depth, achieving accurate genotype calls despite a ∼10% error rate. Our study builds on their findings by scaling up the sample size and applying PacBio HiFi sequencing, which offers lower error rates (HiFi reads >Q20) and highly accurate SV detection (Wenger et al. 2019). Accurately identifying and selecting lines carrying the QTL of interest is the main challenge for plant breeders applying marker-assisted selection (MAS). Typically, SNP markers are used for this purpose. However, low-density markers can miss causative variants or regions closely linked to the QTL of interest, thereby reducing selection accuracy (Bernardo 2008). This is particularly problematic in regions of the genome with high recombination rates or complex SVs, where linkage between markers and causative genes can break down. However, short reads struggle to resolve complex genomic regions and capture SVs. Long-read low-pass sequencing offers a solution. While lower coverage reduces sequencing costs, long reads enable superior genome mapping, capture SVs, and resolve complex regions. This comprehensive view of the genome is particularly valuable for resolving complex QTLs and improving selection accuracy in plant breeding programs.

Our results show that LRLP sequencing is both scalable and effective in capturing a wide range of variant types. The absence of PCR amplification in the long-read workflow eliminates amplification bias—a major limitation in short-read data. Furthermore, our findings are supported by recent work from Magner et al. (2024), who demonstrated improved SNP and large-SV detection by combining short-read data with 4x long-read coverage. Thus, LRLP is a promising and versatile approach for population-scale genomic profiling. Importantly, large-scale execution reveals that LRLP can outperform short-read platforms for variant discovery, especially when SVs play a key role in trait architecture.

### Implementation in breeding programs

Our work demonstrates that LRLP sequencing using PacBio HiFi reads can now be feasibly integrated into routine breeding workflows. While cost remains a consideration, the reduced depth requirements and enhanced accuracy still make LRLP significantly more affordable than traditional high-coverage long-read sequencing. Even when comparing allele calls from LRLP data aligned to a single-reference genome with those from SRLP data aligned to a fully constructed 18-parent pangenome graph, LRLP called more alleles, underscoring its superior resolution and utility, even in resource-constrained settings.

A parallel validation using the same founders genotyped against a linear reference at identical depth provided useful context. Linear-reference genotyping achieved higher callability (50.3%) and allele–identity accuracy (98.7%), with substantially lower allele dropout (11.8% vs 24.0%), reflecting that biallelic SNP calling against a fixed reference requires less per-site coverage than multi-allele pangenome haplotype resolution. However, the linear pipeline cannot detect the structural variants, multi-allelic sites, and founder-specific sequences that represent the core advantage of the pangenome approach. The performance differential therefore reflects an inherent trade-off between per-call confidence at low coverage and the breadth of detectable variation.

Using the pangenome founders as a ground truth set, 1x pangenome genotyping achieved an allele–identity accuracy of 92.2% among co-called sites, with allele dropout and heterozygous sites reduced to homozygous by single-read sampling accounting for most of the discordance rather than outright miscalling (Supplementary Table 2). A parallel linear-reference validation yielded higher callability (50.3%) and allele–identity accuracy (98.7%), reflecting the reduced complexity of biallelic SNP calling relative to multi-allele pangenome haplotype resolution; however, the linear pipeline cannot detect the structural variants and founder-specific sequences that motivate the pangenome approach. Together, these results confirm that 1x LRLP genotyping, while sparse, produces accurate calls suitable for downstream genetic analyses.

Long-read low-pass can be deployed in multiple contexts within breeding programs:

- Late-generation selection: Breeders may use short-read skim-seq in early generations for cost efficiency and apply LRLP in later generations, where fewer lines need testing and selection accuracy becomes paramount.
- Founder line characterization: A single round of LRLP sequencing on founder lines can provide high-resolution data critical for cross-planning and serve as a more cost-effective alternative to full-genome assemblies.
- SNP panel development: LRLP could also be used once to produce a superior SNP panel—an upgrade over short-read WGS—for downstream assays and MAS.

Overall, LRLP offers plant breeders a flexible, accurate, and scalable tool for improving MAS strategies.

Several limitations of this study should be considered when interpreting our results. First, at approximately 1x depth of coverage, sequencing will rarely resolve the genotypic state at heterozygous loci; instead, only the allelic state of a single haplotype is captured. While this limitation applies to both LRLP and SRLP at low coverage, it is particularly relevant for early-generation breeding populations where heterozygosity is high. In situations where heterozygosity needs to be resolved, increasing coverage to at or above the ploidy level will work, as long as long-read sequencing is uniform. While it was beyond the scope of this study, LRLP will also benefit from integration with population-level imputation approaches.

Second, the SV case study presented here, the TSWV resistance-associated insertion on Chr.01, was selected because it is a known insertion present in a founding parent of the MAGIC population, and we wanted to use a genotype-based selection strategy for this trait without requiring phenotypic data. While this provides a compelling demonstration of LRLP's sensitivity for detecting large SVs missed entirely by SRLP, it does not constitute a test of de novo SV discovery. The lack of phenotypic data limits this study's ability to perform trait association mapping, and future work should focus on identifying causally relevant SVs without prior knowledge. In future studies, KhufuPAN could also be used to discover new structural variants rather than just validate known ones. By comparing read-depth patterns and haplotype paths across the pangenome, the pipeline can highlight regions that differ among lines and may represent insertions, deletions, or other rearrangements. Variants that show consistent presence–absence patterns across individuals can then be tested against trait data to confirm their potential functional relevance. Because KhufuPAN produces a population-wide variant matrix, these genotype calls can be paired directly with phenotypic measurements for standard correlation or association analyses.

Along with greater variant density, LRLP exhibited nonsignificantly greater missingness (71.5%) than SRLP (63.7%) only at normalized 1x depth. The non-downsampled sites showed less missingness in LRLP than SRLP. This difference primarily reflects the effects of long-read downsampling rather than inherent platform limitations. Because long reads are fewer and span large genomic regions, reducing coverage removes entire molecules and creates wider gaps, whereas the redundancy of short reads maintains more uniform site-level depth after downsampling. For downstream analyses, moderate levels of missing data primarily reduce statistical power rather than bias the results, and standard genotype–imputation tools can be used to recover missing genotypes. In LRLP datasets, long-range read continuity and more accurate haplotype phasing are expected to enhance imputation accuracy.

While LRLP recovered substantially more variants than SRLP, the practical value of increased variant density depends heavily on the breeding context and local recombination patterns. In situations where enhanced density provides clear advantages—such as fine mapping within regions of high recombination, identification of causative structural variants, or modeling known QTL as fixed effects in genomic selection models—LRLP's broader variant coverage offers meaningful resolution gains. By contrast, in low-recombination regions where variants are in high linkage disequilibrium, marker redundancy offers little additional predictive power over standard SRLP-based marker sets, and the two platforms perform comparably for general genomic prediction. Thus, the major advantage of LRLP lies in discovering and characterizing structural and presence–absence variants, offering improved resolution for causal locus identification and marker development in contexts where recombination and variant architecture make denser coverage informative.

Although this study focused on comparing long- and short-read datasets with equivalent low-pass (∼1x) coverage, future analyses could benchmark LRLP against higher-depth short-read sequencing to quantify cost-to-accuracy trade-offs. High-coverage short-read data may deliver highly reliable genotype calls at lower cost, whereas LRLP captures SVs and haplotypes inaccessible to linear mapping, offering complementary advantages for breeding pipelines.

While the focus of this study is on peanut breeding, the potential applications of LRLP sequencing span far beyond this domain. In diploid species with less complex genome architecture, LRLP is expected to perform even more favorably than demonstrated here: lower ploidy reduces allelic ambiguity, simpler repeat landscapes improve read placement, and the absence of homoeologous subgenomes eliminates a primary source of mapping error in polyploids. Microbial genomics benefits from this approach due to the relatively small genome sizes involved. Long-read low-pass provides high-resolution coverage at low cost, improving taxonomic classification, variant detection, and metagenomic analysis (Agustinho et al. 2024). The consistency and quality of SV detection, combined with cost reduction strategies, position LRLP as a transformative technology across agricultural, ecological, and biomedical applications.

## Conclusion

This study demonstrates that LRLP sequencing is a powerful and cost-effective alternative to short-read sequencing for high-resolution trait mapping in peanut breeding programs. Leveraging LRLP enabled significant improvements in genotype calling, particularly for SVs often missed by short reads, and highlighted the importance of these variants for potential use in marker-assisted selection.

Integrating LRLP with pangenomic analysis provides a scalable framework for accelerating genomic analysis across research and applied settings. As sequencing costs continue to fall and graph-based genomic tools mature, LRLP holds immense potential to redefine how genome-wide diversity is captured, interpreted, and used in selection to develop climate-resilient cultivars for diverse, understudied crops.

## Supplementary Material

jkag196_Supplementary_Data

## Data Availability

Sequence data and the pangenome are available on NCBI under BioProject ID PRJNA1420860. Supplementary data can be found at figshare.com at https://doi.org/10.6084/m9.figshare.28143704. Supplemental material available at G3 online.
