# Supplementary material for: Long-read low-pass sequencing enhances variant detection in a peanut MAGIC population
Source: G3 (Bethesda). 2026 Aug 5;16(9):jkag196. doi: 10.1093/g3journal/jkag196 (PMC13535434; doi:10.1093/g3journal/jkag196)
Supplement: Supplementary file 1 [file jkag196_supplementary_data.docx]

Supplementary Figure 1. A histogram of the mean read lengths for each sample in the peanut population sequenced with long-read low-pass methodology.

Supplementary Figure 2. A scatter plot showing the relationship between yield and coverage in long-read low-pass sequencing data. A low yield is directly related to low coverage (r=.84).

Supplementary Figure 3. Depth‑per‑site histogram normalized to represent genomic fraction at depth

(A) Depth‑per‑site histograms for downsampled short‑read (SRLP) and long‑read (LRLP) sample CA.D12.F1.26 (0.5x, 1x, and 2x coverage).

(B) Depth‑per‑site histograms for filtered and unfiltered SRLP and LRLP datasets of differing coverage. CA.D12.F1.26 has a raw average depth of 0.7x and 0.66x for LRLP and SRLP, respectively. HCCA.D1.F1.04 has a raw average depth of 1.76x and 1.85x for LRLP and SRLP, respectively. HL.D1.F1.06 has a raw average depth of 2.5x and 2.56x for LRLP and SRLP, respectively. Plots display the distribution of sequencing depth (x) normalized to the fraction of the genome covered at each depth. Panels separate SRLP and LRLP libraries, while color within each panel denotes either coverage level or filtering state. The X‑axis shows per‑site sequencing depth (1–10x), and the Y‑axis indicates the fractional representation of the genome. Collectively, the figure demonstrates that SRLP data exhibit higher localized depth over fewer regions, whereas LRLP data maintain broader, more uniform coverage across the genome

Supplementary Figure 4. A box plot comparing reads retained after filtering for misalignment, also called confidence of alignment. The long-read sequences show significantly higher confidence of alignment.

Supplementary Figure 5. Overview of the KhufuPAN workflow. The pipeline begins with a bootstrapping phase in which graph-based indices (GBWT, Giraffe, and multi-index files) are derived from a GFA pangenome graph, variants are called by graph deconstruction, and calls are filtered and reformatted to produce a bootstrapping dataset. In the library processing phase, reads from each library are independently mapped to a personalized graph, variants are called, and filtered call and depth files (.txt call;dep) are generated. Subsets of libraries are then depth-filtered and combined into subset Panmap (.panmap) files. Finally, all subset Panmap files are merged into a single population-level Panmap using KhufuEnv.

Supplementary Figure 6. SNP density across the peanut genome in 1 Mb non-overlapping windows. SNP counts per window are shown for long-read (LRLP, blue) and short-read (SRLP, red) populations aligned to the linear reference (bottom; y-axis capped at 400 SNPs/window) and the pangraph (top; y-axis capped at 10,000 SNPs/window). Windows exceeding the cap are displayed at the cap value. Regions with reduced or absent SNP calls in the linear reference (notably Chr05 and Chr15) correspond to pericentromeric regions. The SRLP pangraph alignment shows extensive call deserts across most chromosomes, consistent with the inability of short reads to resolve complex, repetitive nodes in the pangenome graph.

Supplementary Figure 7. Variant overlap within sequencing analyses. (A) Linear SNPs: UpSet plot summarizing the intersections of SNPs detected in the SRLP Linear and LRLP Linear datasets, revealing shared and unique variant counts within the Linear analysis. (B) Pan SNPs: UpSet plot showing SNP overlap between SRLP Pan and LRLP Pan datasets generated from the KhufuPan reference. (C) Linear Indels (2 bp–1 kb): UpSet plot comparing Indel variants identified in SRLP Linear and LRLP Linear datasets. (D) Pan Indels (2 bp–1 kb): UpSet plot showing Indel overlap between SRLP Pan and LRLP Pan analyses. (E) Structural variants (SVs > 1 kb): UpSet plot comparing SVs identified in SRLP Pan and LRLP Pan datasets, illustrating the extent of shared large structural variants.

Supplementary Figure 8. A heatmap showing variant density between seven peanut cultivars (X-axis) that are founders of the 18-parent MAGIC population. This heatmap highlights an example of high variant density in chromosome A02 in IAC322 and Bailey, which have known introgressions from wild peanut (*A. cardenasii*) in the same chromosome.

**Supplementary Figure 9.** An Integrated Genomics Viewer (IGV) image illustrating read coverage and alignment patterns in an 18 kb window for the TSWV resistance–associated insertion on chromosome 01 (NC94022.Chr01: 12.45–13.08 Mb) extracted from NC94022 in control and MAGIC population samples. Shown are alignments of two downsampled control genomes—Tifrunner v2 (TRv2; susceptible) and NC94022 (resistant)—as well as the MAGIC line D1.F1.08 sequenced with long-read low-pass (LRLP) and short-read low-pass (SRLP) approaches. The NC94022 control demonstrates continuous coverage across the 508 kb insertion region, indicating the presence of the TSWV resistance locus. LRLP data for D1.F1.08 display continuous read coverage across the insertion boundaries, consistent with inheritance of the resistance-associated segment. In contrast, SRLP data show a loss of alignment at the insertion site, reflecting the inability of short reads (~150 bp) to span large structural variants. Reads were aligned to a custom extended reference generated from the NC94022 insertion and 500 kb of flanking TRv2 sequence on either side, enabling side-by-side comparison of alignment continuity between LRLP and SRLP datasets

Supplementary Table 1. Quality assessment of genome assemblies for 18 genotypes used for the construction of the pangenome.

| Genome | Complete BUSCO (fabales_odb10) | Total contigs | Contig N50 (Mb) |
| --- | --- | --- | --- |
| C431-1-1 | 5241 (97.7%) | 581 | 55.20 |
| CC812 | 5239 (97.6%) | 638 | 12.63 |
| Florida 07 | 5285 (98.5%) | 336 | 29 |
| GPNCWS 17 | 5241 (97.6%) | 288 | 54.70 |
| Georganic | 5282 (98.4%) | 100 | 55 |
| IAC322 | 5231 (97.5%) | 223 | 54.51 |
| ICG1471 | 5242 (97.7%) | 871 | 23.98 |
| Lariat | 5241 (97.7%) | 433 | 58.35 |
| Marc1 | 5241 (97.6%) | 568 | 41.38 |
| NC94022 | 5285 (98 .5%) | 212 | 28 |
| TifNV | 5285 (98.5%) | 251 | 59 |
| York | 5257 (98%) | 105 | 48 |
| Georgia12Y | 5275 (98.3 %) | 644 | 13 |
| CC477 | 5285 (98.5%) | 878 | 35 |
| C99R | 5260 (98%) | 110 | 51 |
| CC41 | 5281 (98.4%) | 1121 | 5 |
| NMVal Hap 1 | 5284 (98.5%) | 432 | 14 |
| Bailey | 5247 (97.8%) | 615 | 15 |

Supplementary Table 2. Per-founder 1x genotyping performance for pangenome-based and linear-reference pipelines. All values are percentages of co-called sites (sites that are non-missing in both the 1x and full-depth datasets for that sample). Allele-identity accuracy excludes zygosity errors (dropout and overcall) from the denominator. NMValA and CC477 are outliers in both pipelines, driven by high background heterozygosity rather than pipeline error. Callability = co-called/full-depth called, restricted to sites shared between both filtered datasets. Concordance = exact allele match/co-called. Dropout = full-depth heterozygous called as homozygous at 1x/co-called. Wrong allele = both homozygous but discordant allele/co-called. Allele-identity accuracy = concordant/(co-called − dropout − overcall). Overall callability row is the unweighted mean across founders. Overall values are pooled across all co-called site-sample observations.

|  | Pangenome | | | | | Linear | | | | |
| --- | --- | --- | --- | --- | --- | --- | --- | --- | --- | --- |
| Founder | Callability (%) | Concordance (%) | Droput (%) | Wrong Allele (%) | Allele ID (%) | Callability (%) | Concordance (%) | Droput (%) | Wrong Allele (%) | Allele ID (%) |
| Bailey | 31.8 | 73 | 20.9 | 5.1 | 92.3 | 49.4 | 93.2 | 5.8 | 0.7 | 99 |
| C431 | 26.2 | 72 | 22.5 | 4.9 | 92.9 | 49.2 | 92.4 | 7 | 0.4 | 99.3 |
| C99R | 17.1 | 76.7 | 23.2 | 0.1 | 99.8 | 43.9 | 93.9 | 6.1 | 0 | 100 |
| CC41 | 34.9 | 79.1 | 14.5 | 4.8 | 92.6 | 49.7 | 93.9 | 5.1 | 0.6 | 99 |
| CC477 | 37.8 | 54.6 | 39.9 | 4.7 | 90.9 | 55.2 | 59.6 | 38.2 | 1.5 | 96.5 |
| CC812 | 35.4 | 74.2 | 18.6 | 5.7 | 91.2 | 50.6 | 93.5 | 5.9 | 0.4 | 99.3 |
| Florida07 | 25.4 | 80.1 | 10.8 | 7.2 | 89.8 | 47.3 | 94.8 | 4 | 0.7 | 98.7 |
| GA12Y | 36.1 | 65.1 | 29.2 | 4.3 | 91.8 | 52.2 | 86.3 | 12.7 | 0.5 | 98.8 |
| GP-NC-WS17 | 32.2 | 70.1 | 24.8 | 4.5 | 93.1 | 49.9 | 92.7 | 6.3 | 0.7 | 98.9 |
| Georganic | 33.4 | 76.2 | 18.3 | 4.3 | 93.3 | 52.1 | 93.7 | 5.4 | 0.4 | 99.1 |
| IAC322 | 35.3 | 73.1 | 21.4 | 4.3 | 93 | 50.5 | 93.2 | 5.7 | 0.6 | 98.8 |
| ICG1471 | 24 | 80.7 | 11.1 | 6.7 | 90.7 | 47.6 | 92.7 | 5.9 | 0.8 | 98.5 |
| Lariat | 30.9 | 73.8 | 20.9 | 4.4 | 93.3 | 50.1 | 92.4 | 7.2 | 0.2 | 99.6 |
| 1-Mar | 31.4 | 66.2 | 28 | 4.8 | 91.8 | 49.1 | 86.1 | 13.4 | 0.3 | 99.4 |
| NC94022 | 19.3 | 83.1 | 16.7 | 0.2 | 99.8 | 46.2 | 93.7 | 6.3 | 0 | 100 |
| NMValA | 37.2 | 42.5 | 48.1 | 7.8 | 81.9 | 54.9 | 44.5 | 50.3 | 3.5 | 89.6 |
| TifNV | 37 | 75 | 19.3 | 4.2 | 93 | 56.7 | 92.9 | 6.2 | 0.3 | 99.1 |
| Overall (pooled) | 31 | 70.1 | 24 | 4.8 | 92.2 | 50.3 | 87.1 | 11.8 | 0.7 | 98.7 |

Supplementary Table 3. The average impact score as determined by the software program snpEff of all variants of the indicated length for all samples in the MAGIC peanut population.
